# All I Need Is Two: The Clinical Potential of Adding Evaluative Pairing Procedures to Cognitive Behavioral Therapy for Changing Self-, Body- and Food-Related Evaluations

**DOI:** 10.3390/jcm10204703

**Published:** 2021-10-14

**Authors:** Georg Halbeisen, Georgios Paslakis

**Affiliations:** University Clinic for Psychosomatic Medicine and Psychotherapy, Medical Faculty, Campus East-Westfalia, Ruhr-University Bochum, Virchowstr. 65, 33312 Luebbecke, Germany; georgios.paslakis@rub.de

**Keywords:** conditioning, implicit attitudes, explicit attitudes, cognitive behavioral therapy

## Abstract

Pairing procedures are among the most frequently used paradigms for modifying evaluations of target stimuli related to oneself, an object, or a specific situation due to their repeated pairing with evaluative sources, such as positive or negative images or words. Because altered patterns of evaluations can be linked to the emergence and maintenance of disordered cognitions and behaviors, it has been suggested that pairing procedures may provide a simple yet effective means of complementing more complex intervention approaches, such as cognitive behavioral therapy (CBT). Here, we summarize recent studies that explored the clinical potential of pairing procedures for improving self-esteem, body satisfaction, and food and consumption preferences. While no study has yet combined pairing procedures with CBT, there are several successful examples of pairing procedures in clinically relevant domains and clinical populations. We discuss potential sources of heterogeneity among findings, provide methodological recommendations, and conclude that pairing procedures may bear clinical potential as an add-on to classical psychotherapy.

## 1. Introduction

Cognitive behavioral therapy (CBT) is one of the most widely adopted treatments for mental disorders worldwide [1]. Addressing distorted beliefs, maladaptive expectations, and other disordered cognitions, CBT has been proven successful for treating a variety of common conditions, including phobias [2], depression [3], and personality disorders [4]. However, despite CBT’s demonstrable efficacy [5], remission rates can be unsatisfactory [6,7], or have been shown to decline over time [8]. For example, remission rates for eating disorders, which pose one of the highest mortality risks among mental disorders [9,10], can be as low as 30% [6]. As a result, interest in augmenting CBT using novel technologies and intervention procedures has increased [1,11,12,13,14]. Here, we review recent efforts in using pairing procedures as a potential candidate for complementing CBT. Pairing procedures consist, in essence, of repeatedly pairing two stimuli: a specific target stimulus related to oneself, an object, or a specific situation, and an evaluative source, that is, a stimulus of positive or negative valence. By repeatedly pairing the two stimuli, the procedures aim at changing well-learned evaluations relevant to the emergence and maintenance of disordered cognitions and behaviors (rather than changing disordered cognitions and behavior directly), such as negative self-concepts [15], body ideals [16], or food preferences [17]. Because well-learned evaluations are persistent [18] and can be difficult to change through cognitive interventions such as those used during CBT [19,20], pairing procedures promise a simple yet effective means of complementing CBT and other existing cognitive intervention procedures.

However, the clinical potential of pairing procedures remains mostly unexplored. Few studies have investigated pairing effects in clinically relevant domains, with fewer studies involving clinical populations. No study has yet combined pairing procedures with CBT or other psychological interventions. Moreover, inconsistencies across findings raise validity concerns, which may deter further investigations. To address these concerns and facilitate future research, here, we introduce, summarize, and critically discuss the existing evidence on the effects of pairing procedures in clinically relevant domains regarding food intake, body satisfaction, and self-esteem. Considering methodological and conceptual challenges, we will argue for the relevance of pairing procedures and for further exploring their potential for complementing CBT and other interventions.

## 2. The Clinical Potential of Pairing Procedures

What people like and dislike plays an essential role in cognition and behavior: Evaluations guide how we interpret ambiguous information [21] and influence approach and avoidance behavior [22]. Consistent with this idea, an increasing number of studies show that altered evaluations link to disordered cognitions and behaviors. For example, a generalized negative self-evaluation, i.e., negative self-esteem, both characterizes and predicts the onset of depressive symptoms [23,24,25]; shifted evaluations of body ideals and foods are associated with the development and maintenance of eating disorders [26,27,28,29,30,31]; phobic patients show evaluative biases in processing fear-relevant stimuli and situations [32,33]. These and similar evaluations need not be consistent with an individual’s explicit beliefs or goals (e.g., people frequently desire sweets despite knowing the benefits of restraint [34]) and are less susceptible to conscious introspection and control [19]. Thus, modifying evaluations may offer novel intervention opportunities, which is especially important given that a sizeable number of patients may be unable to engage in CBT due to cognitive impairments [35], insufficient language skills [36], or situational circumstances [37].

**Figure 1:**
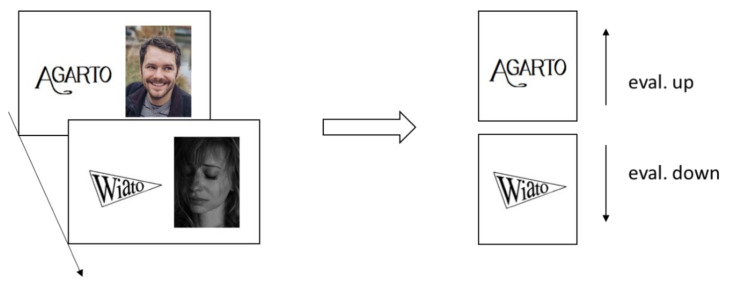
Illustration of a prototypical pairing procedure using (artificial) brand names as target stimuli, and positive and negative images as source stimuli [42]. The repeated pairings of target and source stimuli typically lead to changes in the evaluation of the targets. Targets paired with positive sources are evaluated more favorably than targets paired with negative sources.

Pairing procedures are among the most frequently used paradigms for adjusting evaluations [38,39]. Based on associationism principles [40], these procedures repeatedly present target stimuli, called conditioned stimuli, with liked or disliked source stimuli, called unconditioned stimuli. In a prototypical study, stimuli such as artificial brand names are repeatedly presented for a brief duration with either liked or disliked pictures (see Figure 1). The pairings typically lead to changes in the evaluation of the targets, such that targets paired with liked sources are evaluated more favorably compared to both unpaired targets and targets paired with disliked sources [41].

Numerous studies replicated the “transfer” of valence from source to target using a wide range of stimuli [43], procedural variations [42], and within a range of populations, including toddlers [44] and preschool children [45,46,47]. Moreover, the effects of pairing procedures are temporally stable [48] and do not diminish after repeated target presentation (that is, the effects are resistant to extinction) [18], suggesting that studies could adopt pairing procedures for a range of clinical applications.

Historically, research on pairing procedures has been primarily concerned with how to explain pairing effects on evaluations. For example, early accounts conceived pairing effects as driven exclusively by associative learning mechanisms that operate “implicitly”, that is, outside of an individual’s awareness and without effort or intention [49,50]. Hence, early accounts assumed that merely registering the co-occurrence of the two stimuli on a fast, pre-verbal level creates a memory link between stimulus representations, leading to an activation of the source’s evaluation whenever encountering the target. Although recent methodologically advanced studies provide compelling evidence that pairing procedures are capable of exerting effects with minimal awareness [51], under conditions of reduced processing resources [52], and unintentionally [53], the primacy of implicit processes in explaining pairing effects has since been contested. Specifically, a plethora of studies (for a meta-analysis, see Hofmann et al. [41]) now show that pairing effects depend on selectively attending to the paired stimuli [54], that pairing effects increase with a heightened awareness of the pairings [55] and processing resources [56], and that goal states and motivation modulate pairing effects [57,58]. Moreover, memory manipulations can change pairing effects retroactively [59], and their expression and acquisition are partly controllable [60]. As a result, despite their procedural simplicity, pairing effects likely reflect a mixture of automatic (implicit) and consciously controlled forms of learning, such as hypothesis testing and validation, whose contribution may vary depending on individual and context conditions [61,62].

Nevertheless, inspired by the idea of changing evaluations implicitly, recent years have witnessed a surge of transferring pairing procedures into more applied research areas. For example, pairing procedures were used as an experimental framework to illuminate the mechanisms of brand placement and corporate co-sponsorship in marketing research [63,64]. The majority of examples, however, are found in health and other clinically relevant domains. In the following section, we summarize and discuss the findings of studies that explored the use of pairing procedures as means of therapeutic intervention. Specifically, we critically discuss studies focused on improving self-esteem, enhancing body satisfaction, and changing food-related preferences and behavior. The summarized studies were identified by initial searches in PubMed and PsychInfo databases using the search terms “evaluative conditioning” in conjunction with “self-esteem”, “body image”, and “food preferences”, followed by manual searches in references of selected articles, and citation-based searches.

## 3. Improving Self-Esteem

Initial evidence for the clinical potential of pairing procedures comes from studies interested in raising self-esteem. Based on the idea that self-esteem reflects the evaluations of self-associated stimuli, Baccus et al. [15], Dijksterhuis [65], and Riketta et al. [66] investigated in non-clinical student samples whether pairing self-relevant words (e.g., one’s initials, attributes from a self-descriptive questionnaire, or self-referencing pronouns) with pictures of smiling faces or positive trait words would increase self-esteem. Whereas Baccus et al. presented self-relevant words briefly, but supraliminally, Dijksterhuis and Riketta et al. opted for subliminal target presentation. The decision to present targets subliminally followed the above-mentioned initial conception that pairing procedures operate at an implicit level [65], and, respectively, that an unobtrusive presentation counteracts noticing the attempted self-esteem manipulation and any resulting defensive reactions [66]. To further obscure the research intentions, these studies also tasked participants with non-evaluative categorizations, such as locating the on-screen appearance of source or target stimuli [15,66] or identifying the source’s linguistic status (i.e., word vs. non-word [65]). To assess effects on self-esteem, Baccus et al. and Dijksterhuis used reaction time (RT)-based (i.e., “implicit”) measures of self-evaluations. In contrast, Riketta et al. relied on self-reported (i.e., “explicit”) measures of state self-esteem. The studies found that self-esteem increased relative to control conditions in which the same self-relevant targets were presented but not systematically paired with positive sources.

Following these initial demonstrations, Grumm et al. [67] investigated whether subliminal pairing procedures are differentially effective at changing implicit and explicit measures of self-esteem. Based on three experiments using the Dijksterhuis [65] procedure with a non-clinical sample of undergraduate students, explicit and implicit measures were equally affected by the procedure as long as participants had been instructed to focus on their feelings towards themselves during the measurement rather than on their self-knowledge. The finding suggests that pairing procedures may change self-related evaluations without incurring changes in an individual’s self-relevant beliefs.

Building on the previous success of pairing procedures, Franklin et al. [68] designed the first mobile intervention to reduce non-suicidal, self-injury behaviors linked to lowered self-esteem [25]. Across three experiments, participants with recent and severe histories of self-injuries in the intervention condition observed (supraliminal) pairings of self-related words with positive images and self-injury-related images with negative images. Participants assigned to the control condition only viewed the pairings of neutral images. The specific pairings were presented once at the start of the procedure and then had to be identified over multiple trials among an array of images of different valence. Participants earned points for each correctly identified pair. Using structured interviews and questionnaires, the intervention condition reduced self-cutting episodes, suicide plans, and suicidal behaviors compared to the control condition. However, the effects were not maintained at a 1-month follow-up.

With similar intentions, another recent study by Masuyama et al. [69] investigated the use of pairing procedures for raising low self-esteem as a means of alleviating depressive cognitions. Using the Dijksterhuis [65] procedure, participants from a non-clinical undergraduate student sample were either assigned to a (subliminal) self with positive words or self with neutral words condition. Depressive cognitions were measured 24 h after the intervention using the Depression and Anxiety Cognition Scale [70]. In addition, the study measured self-esteem implicitly immediately after the intervention using an RT-based categorization task. Consistent with previous findings, self-esteem improved in the intervention compared to the control condition. Contrary to the authors’ expectations, however, no intervention effects on overall depressive cognitions were found, which the authors attributed to the significant 24 h depression assessment delay.

Two other studies were unable to replicate pairing effects using the Dijksterhuis [65] procedure. Fleming et al. [71] attempted to change internalized homonegativity in a sample of gay men by pairing self-pronouns (“I”) or “gay” as targets with positive words as sources (or with neutral words in the control conditions). However, neither explicit nor implicit measures of homonegativity and self-esteem revealed the expected effects, which the authors discussed concerning the subliminal presentation procedure. Similarly, Versluis et al. [72] attempted to bolster self-esteem as a means of reducing cardiovascular stress reactivity in a sample of high-worrying students. However, across three experiments, neither explicit nor implicit measures of self-esteem, nor physiological measures of cardiovascular reactivity, revealed an effect of the pairing procedure. Summarizing their efforts, the authors conclude that the results do not support the use of (subliminal) pairing procedures as an intervention.

## 4. Enhancing Body Satisfaction

Inspired by the initially promising evidence for effects on self-esteem, another line of studies investigated the effects of pairing procedures on enhancing body satisfaction, which is critically related to the emergence and maintenance of eating disorders [26]. Specifically, Martijn et al. [16] adapted the procedure of Baccus et al. [15] for a study with female undergraduate students. In a first session, the researchers took three full-body photos of participants in standardized clothing. In a second session, Martijn et al. then paired the photos as targets with pictures of smiling faces as sources and images of other bodies with frowning and neutral faces. Both the participant’s and others´ photos were randomly paired with smiling, frowning, and neutral faces in the control condition. As dependent variables, self-reported body satisfaction (using a self-developed, two-item scale) and (explicit) state self-esteem were assessed. Consistent with the idea that pairing images of one’s own body with positive images improves one’s body image and satisfaction, participants in the intervention condition reported increased levels of body satisfaction.

Extending upon the findings of Martijn et al., Aspen et al. [73] investigated whether they could replicate pairing effects in a sample of female students at risk of developing an eating disorder. At-risk status referred to a score of >47 on the Weight Concerns Scale [74]. Compared to a waitlist control condition, participants in the intervention condition showed an immediate increase in self-esteem, decreased weight and shape concerns, and self-reported restrictive eating, as measured by the Eating Disorder Examination-Questionnaire [75]. Except for self-esteem and restrictive eating, participants maintained improvements in weight and shape concerns at a 12-week follow-up. These preliminary findings may appear promising because body satisfaction can be difficult to change through stand-alone interventions [19,76].

Complementing their initial demonstration, Martijn et al. [77] further explored whether pairing procedures that target evaluations of body comparison standards would indirectly increase body satisfaction. Across two experiments with female undergraduate students, images of supermodels were paired within a categorization task with synonyms of “fake”. In contrast, the procedure paired images of normal-weight models with synonyms of “real”. Compared to a control condition that paired the model categories with neutral nouns and verbs, the intervention condition reduced thin idealization as measured by an Implicit Association Test and improved body satisfaction as measured by the Body Image States Scale [78]. Selimbegovic et al. [79] recently obtained similar findings on indirect effects of pairing procedures. The study asked female undergraduate students to categorize thin-, large-, and beauty-related words. Compared to a control condition in which large and thin were paired equally often with beauty, pairing large more often with beauty than thin reduced self-reported body anxiety [80].

Other studies, however, provide less consistent evidence for the positive effects of pairing procedures. For example, in an earlier study, and using the procedure developed by Riketta et al. [66], Svaldi et al. [81] investigated the effects of subliminally pairing self-referent pronouns (targets) with positive words (sources) on self-esteem and body satisfaction in women before and after mirror exposure. Compared to a control condition that paired self-referent words with negative targets, implicit self-esteem increased, as measured using the Name Letter Task [82]. On the other hand, the study obtained no direct effects on a self-constructed scale of body satisfaction. However, the positive pairing condition bolstered against the negative impact on body image caused by briefly looking into a mirror. This effect is often found in non-clinical populations [19].

In a further direct exploration of the clinical potential of pairing procedures, Glashouwer et al. [83] adapted the Martijn et al. [16] procedure for an online study with adolescent girls who underwent treatment for eating disorders (i.e., anorexia and bulimia nervosa). Two full-body photos of participants dressed in their favorite clothing, taken at a pre-test session, were paired as targets with smiling faces as sources. Compared to the control condition that paired body pictures with themselves, the intervention condition found no effect on self-reported body satisfaction, weight and shape concerns, or general self-esteem. The authors partly attribute the lack of effects to the source stimuli not being positive enough and target pictures with non-standardized clothing.

Attempting to replicate the original Martijn et al. [16] findings, Glashouwer et al. [84] then ran another laboratory study with female undergraduate students. Full-body images of students dressed in black or pink shirts were paired as targets with smiling faces as sources whose location needed to be categorized. Compared to the control condition, whose body images were paired equally often with smiling, neutral, and frowning faces, participants rated their own photographs more positively due to the intervention. The effect, however, was not found to generalize to body satisfaction, as measured by a self-developed scale and the Body Image States Scale [78].

Finally, Kosinski [85] developed an app-based pairing procedure based on Martijn et al. [16] that incorporated several changes for increasing the procedure’s effect. Specifically, the targets were photographs of participants´ bodies or faces (rather than full-body images). Multiple smiling faces were presented simultaneously and sequentially as sources (instead of a single smiling face presented sequentially). Additionally, the procedure asked participants to identify and memorize specific target and source pairs, similar to Franklin et al. [68], rather than merely localizing the source. Despite these changes, however, female undergraduate students in the intervention condition showed no evidence of improved body satisfaction compared to participants in the control condition. The author largely attributed the failure to replicate previous findings to the recruitment of a non-clinical sample, which may have had a high body satisfaction to begin with, rendering it challenging to detect any further improvements.

## 5. Changing Food-Related Preferences and Behavior

Studies on food and consumption preferences, which link to disordered eating [86], found more consistent evidence for the clinical potential of pairing procedures. For example, Dwyer et al. [87] conducted two laboratory experiments with undergraduate students that paired images of fruits and vegetables as targets with body images as valence sources. Specifically, images of obese bodies served as negative sources, whereas images of normal-weight bodies served as neutral controls. The study presented all stimuli supraliminally and instructed participants to attend to and memorize the pairings. Ratings of food liking revealed that foods paired with obese body shapes were rated less favorably than foods paired with normal-weight body shapes. Hollands et al. [17] replicated these findings in a student sample with snacks rather than healthy foods as targets using an almost identical procedure. The study found effects on both explicit and implicit measures of food evaluation as well as in a behavioral choice test (for further replications with non-clinical, all-female samples, see Lebens et al. [88] and Wang et al. [89]).

Another set of studies by Walsh and Kiviniemi [90] and Hensels and Baines [91] provide converging evidence for the positive effects of pairing procedures on food preferences and choice behavior (see also Ebert et al. [92] and Mattavelli et al. [93]). Differing from previous investigations, both studies presented healthy foods as targets with positive or neutral food-unrelated stimuli as sources (for a similar study in preschool children, see Halbeisen and Walther [47]). Moreover, pairings were embedded within a stream of other stimuli to conceal the studies´ intentions, although the study instructed participants to attend to the screen carefully. Across studies, healthy food choice increased as a function of positive stimulus pairings. Bui and Fazio [94] later showed that such pairing effects also generalized to other healthy foods beyond the specific targets as long as the instructions rendered health a salient attribute during target presentation.

In a further extension of this line of research, Houben et al. [95] found that pairing beer-related pictures as targets with negative words and pictures as sources decreased evaluations and consumption of beer in an undergraduate cohort compared to the control condition with no exposure to systematic pairings (for similar findings on soft drink consumption, see Shaw et al. [96]). Similarly, Zerhouni et al. [97,98] showed that pairing images of alcoholic beverages as targets with negative images as sources reduced undergraduate students’ purchase intentions for alcoholic beverages, compared to a control condition that paired beverages with neutral images.

However, there are also inconsistencies despite the initial supporting evidence (see also Masterton et al. [99]). For example, Hollands and Marteau [100] attempted to replicate earlier findings on food choice [17] in a general population sample in an online study but did not find an effect of pairings. Instead, all participants exposed to images of unhealthy eating consequences (e.g., images of obese bodies) showed improvements in healthy food choices. At the same time, one should note that target valence, different from all previously cited studies, was manipulated between rather than within participants. As such, this study was unable to distinguish between pairing and exposure effects.

Finally, in a study with young adults, Alblas et al. [101] found that pairing effects on improving healthy food choice depended on pre-existing positive evaluations. Similarly, Haynes et al. [102] found more pronounced pairing effects in individuals with low inhibitory control. The finding suggests that differences in the personal relevance of source or target stimuli could moderate the effect, which could be particularly valuable for treating specific eating disorders [103], but which could also limit the application of pairing procedures.

## 6. Discussion

Pairing procedures may be a simple yet effective means of complementing CBT by changing well-learned evaluations relevant to the emergence and maintenance of disordered cognition and behavior. As shown above, there are several examples, including with clinical populations, of randomized controlled studies that reveal promising effects of pairing procedures for improving self-esteem, body satisfaction, and food and consumption preferences. However, no study has yet combined pairing procedures with CBT, and there is considerable heterogeneity among findings, both within and across study domains. As some authors suggest, the failures to replicate pairing effects could be due to inadequate choice of stimuli or controls [71,83,100], statistical “ceiling effects” in non-clinical populations [85], or extraneous influences when measuring pairing effects with a significant temporal delay [69]. However, replication failures may also raise more profound concerns about the validity of pairing procedures [72] and ultimately discourage further explorations of their clinical potential. We will discuss potential sources for heterogeneous findings of pairing procedures in clinically relevant domains to address these concerns and facilitate future research.

### 6.1. Subliminal Stimulus Presentation

Arguably, the appeal of using pairing procedures in clinically relevant domains originates from their initial conception as “implicit” modes of intervention that do not require any conscious processing of target and source stimuli [49,50]. Consistent with this idea, we identified several examples of pairing procedures, particularly among studies on raising self-esteem, that actively attempted to preclude conscious processing by presenting stimuli briefly [65,67,69] or otherwise subliminally [66].

A subliminal presentation may have the added benefit of preventing defensive reactions in response to a perceived manipulation attempt [66] but is also a likely source of heterogeneity among findings. Specifically, and despite continuing descriptions of pairing procedures as implicit process interventions [30,104,105], it has been well-established that pairing effects reflect a mixture of implicit and consciously controlled learning processes, the distinct contribution of which may vary depending on individual and context conditions [61,62]. Limiting learning conditions to only implicit learning processes should therefore reduce the effect’s reliability, especially if one does not consider specific individual and context conditions of implicit processes. Consistent with this notion, many studies show that pairing effects generally benefit from an individual’s explicit processing of the pairings, compared to the low reliability of pairing effects under conditions that only allow for implicit processing [55]. Therefore, future studies, including those on raising self-esteem, may benefit from relying on supraliminal target and source presentation. In fact, Franklin et al. [68] succeeded in raising self-esteem in a clinical population by presenting target and source stimuli supraliminally, a result that may also help to alleviate concerns about defensive reactions.

On a further conceptual note, it is important to stress that recognizing controlled learning processes does not preclude the possibility of “implicit” pairing effects. Since implicit evaluations may very well originate from explicit learning processes [106,107], pairing procedures may nevertheless offer opportunities to modify evaluations that are difficult to control consciously.

### 6.2. Processing Goals

One might also trace another source of heterogeneity among findings to the impact of different processing goals on target and source encoding. Unlike in many pairing procedures from basic research areas [41], participants within a majority of the above-summarized studies did not passively observe the pairings but pursued specific processing goals, such as localizing targets [84], memorizing pairings [87], and categorizing targets according to specific features (e.g., “beauty-related” [79]), or along general stimulus dimensions (e.g., word vs. non-word [65]). These and similar processing goals effectively engage in study participation and focus participants’ attention on stimulus processing.

However, processing goals may also interfere with encoding the source’s positive or negative valence (rather than promoting valence encoding or remaining neutral) and thus deter from obtaining the intended pairing effects [54,108]. For example, Gast und Rothermund found that instructing participants to categorize targets along a valence-unrelated dimension, similar to studies using the Dijksterhuis [65] procedure, eliminated pairing effects compared to a valence-related categorization condition [109]. Other studies observed similar effect modulations, although less extreme in magnitude [57,58]. These effects suggest that future studies on the clinical potential of pairing procedures, especially when intending to use categorization tasks [65,69,72,85], could benefit from closely examining how an induced processing goal might affect source valence encoding.

The impact of processing goals on pairing effects may further depend on structural task characteristics. Compare, for example, Franklin et al. [68], who used app-based pairing procedures to raise self-esteem, with Kosinski [85], who found no improvements in body satisfaction using a similar app-based pairing procedure. Both studies induced similar processing goals unrelated to source valence by instructing participants to identify specific source stimuli. However, Franklin et al. presented to-be-identified positive sources among differently-valenced stimuli within intervention conditions, whereas Kosinski presented to-be-identified positive sources among other positive stimuli. In other words, positive valence was a predictive feature of source identity in Franklin et al.’s study, whereas source valence was an irrelevant feature in Kosinski’s study. Therefore, participants would be inclined to process source valence in Franklin et al. to improve task performance, from which pairing effects would benefit, but to ignore source valence in Kosinski, leading to reduced pairing effects [42,110]. Thus, to predict the effects of processing goals on pairing effects, it might also be necessary to examine their interaction with other task characteristics.

### 6.3. Relative Target Specificity

Varying levels of target specificity relative to the specificity of outcome measurements might also lead to heterogeneity among findings. Consider, for example, the difference in relative target specificity between studies on improving food and consumption preferences, which showed more consistent evidence for pairing effects, and studies on improving self-esteem and body satisfaction. While studies on food and consumption preferences use target stimuli such as pictures of foods or beverages during preference and behavioral measurements [47,87], the specific target stimuli used in studies on self-esteem and body satisfaction, such as pronouns or own body pictures, are not used during measurement. Instead, the studies use more general outcomes, such as depression questionnaires [69] or body image scales [78], to measure pairing effects. Thus, some alleged replication failures of clinically relevant pairing effects could instead indicate the absence of the generalization of pairing effects from specific target stimuli to more general outcome measures [84]. Future studies on pairing effects in clinically relevant domains might benefit from matching the specificity of target stimuli and outcome measures.

Admittedly, in studies exploring the clinical potential of pairing procedures, it might neither be feasible nor desirable to limit outcome measurements to certain levels of target specificity. After all, desirable outcomes would include changes in body satisfaction rather than changes in evaluating a specific body photograph [84]. The consideration of target specificity in future studies on the clinical potential of pairing procedures is still essential, as mismatching levels of target and outcome specificity imply that target generalization may require active encouragement. Bui and Fazio [94] provide an example of active generalization encouragement by having their participants categorize target stimuli according to more general features (i.e., categorizing foods based on healthiness before pairing with different sources). As a result, positive effects of food pairings generalized to overall healthy eating intentions. Thus, *how* targets are processed may not only affect whether pairings are encoded (see Section 6.2 above), but whether their encoding exerts the intended effect on more general outcome measurements.

### 6.4. Standardization, Manipulation Checks, and Dosing

Besides the subtleties of pairing procedures, there are also more general sources for the heterogeneity of findings. Different studies reveal varying degrees of target standardization, ranging from highly standardized pretested images [97] and general pronouns [65] to the peculiarities of photographs of participants in their favorite clothing [83]. Although, feasibility and desirability concerns may limit the use of standardized target and source stimuli for specific applications, for example, when addressing body image concerns (but see [73,77]); it must nevertheless be considered that lower degrees of standardization, and thus objectivity of the procedure, will likely reduce pairing effects’ reliability.

In a similar vein and related to the issue mentioned above of target specificity and generalization, only a few studies conducted manipulation checks. Thus, hardly any study measured pairing encoding independent of the predicted pairing effect. As a result, the summarized studies cannot distinguish between the successful encoding of target-source pairings (i.e., learning) and changes in evaluations and behavior (i.e., the effect of learning). As multiple processes affect evaluations and behavior [104,106], it is not logically warranted to infer an absence of learning from an absence of its predicted effect reversely. Instead, the absence of a pairing effect could indicate that one’s prediction needs to account for yet unidentified moderator conditions. Grumm et al. [67], for example, demonstrated that pairing effects on explicit measures of self-esteem are not generally absent, but can be obtained if participants are given specific rating instructions. Thus, future studies on the clinical potential of pairing procedures may benefit from examining target-source encoding independent of pairing effects, for example, by measuring other indices of associative learning [42].

Finally, the summarized studies show considerable variation in dosing concerning the frequency of pairings and the intensity of evaluative sources. For example, the procedure of Dijksterhuis [65] lasted a total of 30 trials, whereas Martijn et al. [16] had 270 trials and Dwyer et al. [87] had 24 trials. Moreover, some studies used rather intense affective pictures as evaluative sources [95], whereas others relied on the associated evaluations of self-referential pronouns [93]. Although consistent effects between the magnitude of pairing effects and the frequency of pairings or the intensity of evaluative sources were not obtained in previous meta-analyses [41], individual studies suggest the possibility of inverted u-shape relations between effect magnitude and both pairing frequency and source intensity [62,111]. Thus, future studies on the clinical potential of pairing procedures may also be advised to explore the role of these and similar parameters systematically.

## 7. Conclusions

Because pairing procedures can be used to change well-learned evaluations relevant to the emergence and maintenance of mental disorders that are difficult to change through cognitive interventions, pairing procedures have been suggested as a potential candidate for complementing CBT. However, their clinical potential still needs to be explored further, as we were unable to identify any study that combined pairing procedures with CBT or any other psychological intervention procedure. Thus, whether or not the addition of pairing procedures to CBT may improve, for example, remission rates for eating disorders [6], cannot be answered at present. Nevertheless, the available literature includes several examples of successful pairing procedures, including with clinical populations, related to improving negative self-concepts [15], body ideals [16], and food preferences [17]. Given the relevance of these and similar evaluations for the emergence and maintenance of disordered cognitions and behaviors [23,24,25,26,27,28,29,30,31,32,33], pairing procedures may indeed offer a simple yet effective means of complementing CBT.

Qualifying the apparent simplicity, however, heterogeneous findings suggest that the implementation of pairing procedures still needs improvement. Among other issues, we discussed the potential impact of subliminal stimulus presentation, induced processing goals, target specificity and generalization, and standardization as areas that need further consideration. The successful clinical implementation of pairing procedures may require attention to details beyond what has been considered in basic research areas, recognizing procedural and population characteristics [37]. However, given that pairing procedures could be especially relevant for individuals who cannot engage in CBT due to cognitive impairments [35] or insufficient language skills [36], further exploring the clinical potential of pairing procedures appears well-warranted.
